# Molekulare Medizin: Pathobiochemie als Schlüssel zur personalisierten Therapie vererbter Krankheiten

**DOI:** 10.1007/s00112-021-01252-3

**Published:** 2021-07-29

**Authors:** J. A. Mayr, R. G. Feichtinger, M. T. Achleitner, K. Brugger, K. Kutsam, J. Spenger, J. Koch, P. Hofbauer, F. B. Lagler, W. Sperl, D. Weghuber, S. B. Wortmann

**Affiliations:** 1 Universitätsklinik für Kinder- und Jugendheilkunde, Paracelsus Medizinische Privatuniversität, Müllner Hauptstr. 48, 5020 Salzburg, Österreich; 2 Arzneimittelproduktion, Landesapotheke Salzburg, Betrieb des Landes Salzburg, Müllner Hauptstr. 50, 5020 Salzburg, Österreich; 3 Institut für angeborene Stoffwechselerkrankungen, Paracelsus Medizinische Privatuniversität, Strubergasse 22, 5020 Salzburg, Österreich; 4 Amalia Children’s Hospital, Radboudumc, Geert Grote Plein Zuid 10, 6525GA Nijmegen, Niederlande

**Keywords:** Stoffwechselerkrankungen, Whole genome sequencing, Substitutionstherapie, Detoxifikation, Gentherapie, Metabolic diseases, Whole genome sequencing, Therapeutic substitution, Detoxification, Gene therapy

## Abstract

Genetische Defekte werden vielfach noch als Schicksal empfunden, mit dem man sich Zeit seines Lebens abfinden muss. Es stimmt, dass vererbte Anlagen in vielen Fällen zu schweren Krankheiten führen, allerdings stimmt es auch, dass der Anteil von genetischen Defekten, bei denen eine Therapieoption besteht, stetig wächst und sich der Ausbruch von Krankheitssymptomen bei einigen davon bestenfalls gänzlich verhindern lässt. Die Kenntnis des genauen molekularen Krankheitsmechanismus liefert oft die Grundlage für einen Therapieansatz. Zum Auffinden des genetischen Defekts haben die Möglichkeiten der genomweiten Sequenzierung und ihr mittlerweile breiter Einsatz in der Diagnostik entscheidend beigetragen. Nach dem Nachweis einer genetischen Veränderung braucht es aber noch die Untersuchung der pathobiochemischen Konsequenzen auf zellulärer und systemischer Ebene. Dabei handelt es sich oft um einen längeren Prozess, da der volle Umfang von Funktionsausfällen nicht immer auf Anhieb erkennbar ist. Bei metabolischen Defekten kann die Therapie ein Auffüllen von fehlenden Produkten oder eine Reduktion von giftigen Substraten sein. Oft lässt sich auch die Restfunktion von betroffenen „pathways“ verbessern. Neuerdings haben Therapien mit direkter Korrektur des betroffenen Gendefekts Einzug in die therapeutische Anwendung gefunden. Da die ersten Krankheitssymptome in vielen Fällen früh im Leben auftreten, trifft die Kinderheilkunde eine Vorreiterrolle in der Entwicklung von Therapieansätzen.

## Abstract (en)

Genetic defects are often still regarded as a life-long fate, which one has to cope with. It is true that in many cases an inherited disposition may lead to a severe disease; however, it is also true that the number of genetic defects with a treatment option is continuously increasing and in some of them the onset of disease symptoms can even be totally prevented. Knowledge of the precise molecular pathomechanism is often the basis for a treatment concept. Genome-wide sequencing has tremendously increased the possibility to identify a genetic defect and its broad application has meanwhile made a decisive contribution in routine diagnostics. After identifying a genetic alteration, it is still necessary to investigate the pathobiochemical consequences on the cellular and systemic level. This can be a time-consuming process since not all functional consequences can be immediately recognized. In the case of metabolic defects the treatment strategy can either be a supplementation of missing products or a removal of toxic substrates. The residual function of affected pathways can also often be improved. Recently, the direct correction of the affected genetic defects has become a treatment option for a selected number of diseases. As the first symptoms of disease usually occur early in life, pediatrics has a pioneering role in developing treatment strategies.

## Genetisch bedingte Krankheiten

Derzeit sind in der Datenbank Online Mendelian Inheritance in Man (OMIM, https://omim.org) ca. 4000 Gene gelistet, deren genetische Varianten beim Menschen zu Krankheiten führen. Das heißt, dass bisher bei etwa einem Fünftel der ca. 20.000 Gene, die beim Menschen bekannt sind, krankheitsrelevante Varianten gefunden wurden. Neben den monogenetischen Krankheiten gibt es globalere genetische Defekte, bei denen ganze Chromosomen (z. B. Trisomie 21, Monosomie X) oder Teile davon verändert sind. Genetische Defekte können von einem Elternteil z. B. in dominanter Form oder von beiden Elternteilen in rezessiver Form vererbt sein oder auch bei Patienten neu entstanden sein. Die Auswirkung von genetischen Varianten kann ein kompletter oder nur teilweiser Verlust der Funktion des betroffenen Genprodukts sein. Dementsprechend können unterschiedliche Schweregrade von genetischen Krankheiten festgelegt sein, wobei eine derartige Korrelation von Genotyp und klinischem Phänotyp nicht auf alle genetischen Defekte zutrifft. Wie bei anderen Krankheiten, können auch bei genetischen Defekten zusätzliche Faktoren eine Rolle spielen.

Die Häufigkeit genetischer Krankheiten ist sehr breit gefächert, mit einem Anteil z. B. bis zu 10 % der Bevölkerung mit Glucose-6-phosphatdehydrogenase-Mangel als Ursache des Favismus v. a. in Malaria-Gebieten [5]. In Mitteleuropa zählt die familiäre Hypercholesterinämie zu den häufigsten genetischen Krankheiten mit einer Trägerfrequenz bis zu 1/200 für die dominante Form [18]. Die Mehrzahl der genetischen Krankheiten ist allerdings selten (zystische Fibrose 1/3000, Mitochondriopathien 1/5000, Phenylketonurie [PKU] 1/7000, spinale Muskelatrophie [SMA] 1/10.000), und von vielen genetischen Defekten gibt es weltweit nur einige bekannte Fälle.

## Diagnostik und pathobiochemische Charakterisierung

In der Diagnostik von Kindern mit einer Entwicklungsstörung hat die Molekulargenetik mithilfe der Exom- oder Genomsequenzierung mittlerweile eine tragende Rolle eingenommen. Damit ist es oft vergleichsweise rasch möglich, ein bekanntes krankheitsrelevantes Gen oder ein Kandidatengen zu benennen, das beim betroffenen Kind auffällig verändert ist. Wenn es sich um ein Kandidatengen handelt, bei dem bisher noch kein Patient beschrieben wurde, bedarf es jedenfalls weiterer Abklärung, um sich auf eine pathologische Relevanz festlegen zu können. Am besten wäre es, weitere Patienten mit ähnlicher genetischer und klinischer Auffälligkeit zu finden. Das kann beispielsweise durch Austausch mit Kolleginnen und Kollegen etwa über Plattformen wie GeneMatcher (https://genematcher.org) gelingen.

Wenn ein neuer genetischer Defekt bei einem Patienten gefunden wird, stellt sich auch immer die Frage nach pathobiochemischen Auffälligkeiten auf zellulärer Ebene. Für den Fall, dass das betroffene Gen bereits in grundlagenwissenschaftlichen Arbeiten z. B. in anderen Organismen untersucht wurde, kann versucht werden, die beschriebenen Auffälligkeiten beim Patienten nachzuweisen. Falls es sich um Metaboliten handelt, die in Körperflüssigkeiten nachweisbar sind, können entsprechende Untersuchungen durchgeführt werden. Sind Veränderungen nur in Zellen nachweisbar, können weitere Untersuchungen ggf. an Blutzellen oder Hautfibroblasten von Patienten erfolgen. Für den Fall, dass ein betroffenes Gen gewebespezifisch exprimiert wird, muss ggf. klinisch betroffenes Gewebe im Rahmen einer Biopsie entnommen werden, um pathologische Veränderungen sichtbar machen zu können. Alternativ dazu kann versucht werden, Patientenzellen in andere Zelltypen (z. B. Fibroblasten in Neuronen) zu differenzieren, um Auffälligkeiten nachzuweisen. Komplizierter ist es, wenn die Funktion eines Genprodukts noch weitgehend unbekannt ist. Hier könnten ggf. Techniken weiterhelfen, bei denen möglichst viele Parameter erfasst werden, wie z. B. Metabolomik, einschließlich Glykomik (Glykosylierung von Proteinen und Lipiden) und Lipidomik (wasserunlösliche Metaboliten), sowie auch RNA-Sequenzierung und Proteomik.

Bei neu gefundenen Defekten interessieren die pathobiochemischen Auffälligkeiten auf zellulärer Ebene

Beim anschließenden Vergleich von Patienten- und Kontrollmaterial können Unterschiede erkannt werden, die Rückschlüsse auf die Bedeutung des betroffenen Gens zulassen. Ein geeignetes Modell in einem anderen Organismus kann ebenfalls hilfreich sein; damit kann aber ein erheblicher Aufwand verbunden sein, und die Vergleichbarkeit mit dem Menschen muss nicht immer gegeben sein.

## Von der Pathobiochemie zur Therapie

Für die Bestätigung von Wirksamkeit und Verträglichkeit einer Therapie ist die möglichst präzise Kenntnis des natürlichen Krankheitsverlaufs, am besten über die gesamte Lebensspanne und für unterschiedliche Schweregrade, von großer Bedeutung. In der Praxis stehen bei seltenen Krankheiten allerdings meist nur Daten von kleinen, vielleicht auch noch heterogenen Patientengruppen sowie einem begrenzten Beobachtungszeitraum zur Verfügung.

**Figure 1:**
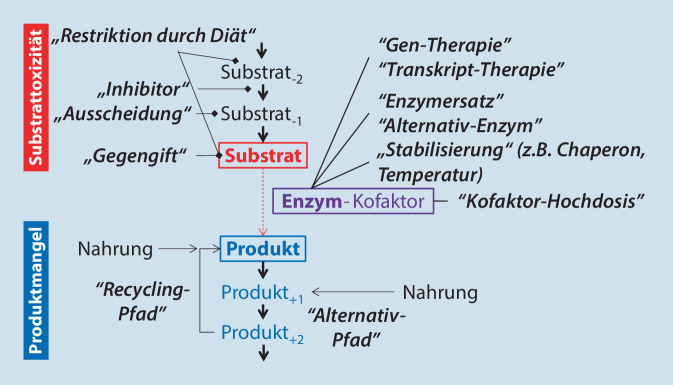

Oft ist die präzise Aufklärung der pathobiochemischen Veränderungen, z. B. mithilfe von Patientenzellen, der Schlüssel für therapeutische Optionen. Im Stoffwechsel wird typischerweise aus einem Substrat unter Zuhilfenahme eines Enzyms ein Produkt gebildet (Abb. 1). Bei unzureichender Funktion des Enzyms kommt es häufig zur Anstauung aufseiten des Substrats und zum Mangel aufseiten des Produkts. Überhöhte Konzentrationen von Ausgangssubstanzen können sich toxisch auswirken; ein Fehlen eines Produkts kann einen Mangel hervorrufen. Daraus ergibt sich eine Reihe von Therapieansätzen, die in folgende 3 allgemeine Gruppen zusammengefasst werden können (Abb. 1):

- *Therapieansatz 1*: Die Konzentration toxischer Substrate kann z. B. durch spezifische Diäten, Inhibition ihrer Synthese und Forcieren ihrer Ausscheidung reduziert werden. Zudem kann toxischen Wirkungen durch „Gegengifte“ spezifisch entgegengewirkt werden.
- *Therapieansatz 2*: Produkte können über die Nahrung oder aus alternativen Stoffwechselwegen zugeführt werden.
- *Therapieansatz 3*: Durch Gen- oder Transkripttherapie kann der zugrunde liegende Defekt möglichst ursächlich korrigiert und die körpereigenen Zellen können zur Synthese des normalen Genprodukts befähigt werden. Fehlendes Enzym kann z. B. durch Verabreichung von synthetischen Formen desselben Enzyms (Enzymersatz) oder ähnlich wirkenden Enzymen (alternatives Enzym) substituiert werden. Vor allem, wenn das betroffene Enzym noch eine relevante Restfunktion zeigt, kann diese durch Kofaktoren gesteigert bzw. das Enzym durch pharmakologische Chaperone vor vermehrtem Abbau geschützt werden.

Stehen für einen der genannten Ansätze Substanzen zur Verfügung, die bereits als Arzneimittel oder Nahrungsergänzungsmittel zugelassen sind, kann der Weg zur Anwendung bei Patienten mit einer (neuartigen) genetischen Krankheiten kurz sein.

## Beispiele für erfolgreiche Therapien bei genetischen Krankheiten

Im Folgenden werden einige Beispiele mit unterschiedlichen Therapiemöglichkeiten v. a. aus dem Bereich des Stoffwechsels aus der eigenen Erfahrung der Autoren präsentiert, aber auch Beispiele für allgemeinere Therapieansätze von genetisch bedingten Krankheiten gegeben.

### Stoffwechselerkrankungen

#### Limitierung von toxischen Substanzen (Beispiele PKU, ECHS1-, HIBCH-Defekt)

Die erste erfolgreiche Therapie einer genetischen Krankheit gelang dem deutschen Pädiater Horst Bickel [3]. Tatsächlich ging man bis zu diesem Zeitpunkt allgemein davon aus, dass genetische Krankheiten nicht kausal behandelt werden können. Die PKU entsteht durch den Defekt der Phenylalaninhydroxylase, codiert durch das *PAH*-Gen. Phenylalanin ist als essenzielle Aminosäure ein wichtiger Bestandteil der Nahrung. Bei einer ca. 5- bis 10-fach erhöhten Konzentration im Plasma wird Phenylalanin allerdings zur toxischen Substanz, bzw. daraus entstehen andere toxische Metaboliten. Die Standardtherapie der PKU besteht darin, dass die Menge an Phenylalanin auf ein Maß reduziert wird, das für die Ausbildung von körpereigenen Proteinen ausreicht und bei dem es zu keiner Toxizität kommt. Diese Phenylalaninreduktion lässt sich durch eiweißarme Diät erreichen, wobei andere essenzielle Aminosäuren zugegeben werden können. Es wird also eine Substratreduktion und eine Produktsupplementierung durchgeführt.

Durch eine spezifische PKU-Diät werden toxische Phenylalaninkonzentrationen vermieden (Therapieansatz 1)

Relativ aktuelle Beispiele mit einem vergleichbaren Pathomechanismus sind der ECHS1- und der HIBCH-Defekt, 2 enzymatische Schritte im Valinabbau, bei denen sich toxische Methacrylsäure anhäuft und die Funktion der Mitochondrien beeinträchtigt wird. Valin ist wie Phenylalanin eine essenzielle Aminosäure und kann somit auch über die Zusammensetzung der Nahrung reguliert werden. Rezente Arbeiten zeigen eine signifikante Verbesserung der Labor- und klinischen Parametern; allerdings hatten alle Patienten bereits vor Therapiebeginn über Monate und Jahre neurologische Defizite ausgebildet [1, 11]. Im nächsten Schritt müsste also gezeigt werden, welche Entwicklung bei Patienten mit ECHS1- bzw. HIBCH-Defekt möglich ist, wenn die valinreduzierte Therapie vor dem Auftreten neurologischer Symptome begonnen wird.

#### Vermeiden von Überlastung eines Stoffwechselwegs (Beispiel PPA2-Defizienz – Ethanol)

Die mitochondriale Pyrophosphatase wird durch *PPA2* codiert und ist für den Phosphathaushalt im Energiestoffwechsel von Bedeutung. Bei allen bisher identifizierten Patienten mit biallelischen *PPA2*-Varianten besteht eine gewisse Restfunktion der Pyrophosphatase, wobei es schwere Verlaufsformen mit Versterben in der Neonatalperiode bis frühen Kindheit gibt, jedoch auch Jugendliche mit plötzlichem Herzversagen nach dem Konsum einer geringen Menge alkoholischer Getränke. Ethanol wird nach der Umwandlung in Essigsäure in Mitochondrien zu Acetyl-Koenzym A aktiviert; hierbei entsteht auch Pyrophosphat. Durch dieses zusätzlich gebildete Pyrophosphat kommt es offensichtlich zu einer Überlastung des mitochondrialen Phosphatmetabolismus und einer Herzinsuffizienz. Ebenfalls betroffene Geschwister, die keinen Alkohol konsumiert haben, erreichten die 4. Lebensdekade ohne schwerwiegende Symptome [7, 12], sodass der Meidung des Substrats Ethanol offenbar eine wichtige Bedeutung zukommt.

#### De-novo-Synthese vs. Recycling (Beispiele Uridin – CAD-Defekt, Fucose – GFUS)

Der menschliche Stoffwechsel ist in vielen Bereichen auf Sparsamkeit ausgelegt. Beispielsweise können Pyrimidine durch Neusynthese oder Recycling von Pyrimidinresten in DNA, RNA und Mononukleotiden entweder aus der Zelle oder aus der Nahrung gebildet werden. Bei Defekten der Neusynthese von Uridinmonophosphat z. B. im multifunktionalen CAD-Enzym kann die Schwere der Erkrankung variieren, je nachdem, wie der Betroffene aus der Nahrung mit Pyrimidin versorgt wird. Säuglinge, die ausschließlich gestillt werden, können früher Symptome wie Entwicklungsretardierung und Epilepsie sowie eine Anisopoikilozytose entwickeln als jene, die Formula-Nahrung erhalten, da Letztere meist mit Uridin angereichert ist. Frühe Diagnose und Substitution mit Uridin dürften der Schlüssel für ein gutes Outcome bei CAD-Defekt-Patienten sein [15, 21].

Ähnlich sieht die Situation bei einem kürzlich gefundenen Defekt der de novo Fucose-Synthese aus. Auch hier kann der Defekt durch Fucosegabe über die Nahrung umgangen werden. Eine 4‑jährige Patientin mit globaler Entwicklungsretardierung holt unter Fucosesubstitution innerhalb eines Jahres merklich in der Entwicklung auf und beginnt zu sprechen [6].

#### Alternativer Weg in der Energiebereitstellung (Beispiele ketogene Diät bei PDHC-, GLUT1-Defekt, Citrindefizienz)

Es gibt eine Reihe von Defekten, bei denen die Bereitstellung von Energie aus Kohlenhydraten über die Glykolyse nicht adäquat funktioniert. Ein klassisches Beispiel ist der Pyruvatdehydrogenase Komplex(PDHC)-Defekt, der am häufigsten durch das X‑chromosomal codierte *PDHA1*-Gen der E_1__α_-Untereinheit des Enzyms verursacht wird. Pyruvat, das Endprodukt aus der Glykolyse, kann nicht zu Acetyl-Koenzym A umgewandelt werden und fehlt für die Oxidation über den Zitratzyklus und die oxidative Phosphorylierung. Alternativ kann Acetyl-Koenzym A aber auch aus der Fettsäureoxidation gebildet werden, wodurch die ketogene Diät das Energieproblem beim PDHC-Defekt auf einem alternativen Weg umgeht und Betroffene sich darunter auf einem stabilen Niveau entwickeln können [24].

Die ketogene Diät umgeht das Energieproblem beim PDHC-Defekt auf alternativem Weg (Therapieansatz 2)

Beim GLUT1-Defekt, bei dem der Glucosetransporter SLC2A1 betroffen ist, der v. a. das Gehirn mit Energie in Form von Glucose versorgt, kann alternativ auf Ketonkörper als Energiequelle zurückgegriffen werden, deren Transport normal funktioniert. Die Epilepsie und Bewegungsstörung dieser Patienten sprechen meist gut auf die Behandlung mit ketogener Diät an [28]. Bei der Citrindefizienz ist die leberspezifische Isoform des Malat-Aspartat-Shuttle, codiert durch *SLC25A13*, betroffen. Bei diesem Defekt ist der Redoxtransport von NADH aus der Glykolyse eingeschränkt. Patienten mit einer Citrindefizienz können sich früh mit einer Hepatomegalie und Leberfunktionsstörung präsentieren. Unter der Therapie mit fettreicher, kohlenhydratarmer Ernährung entwickeln sie sich meist ausgezeichnet. Wichtig ist, dass ein Notfallplan vorliegt und keinesfalls eine Glucoseinfusion verabreicht wird, da die Betroffenen darunter versterben können [22].

#### Detoxifikation (Nitisinon – Tyrosinämie Typ I, Phenylbutyrat – „urea cycle disorders“)

Das Anhäufen von toxischen Metaboliten ist ein häufiges Problem bei Stoffwechseldefekten. Bei Harnstoffzyklusdefekten („urea cycle disorders“, UCD) kommt es oft schon in den ersten Lebenstagen zu einer lebensbedrohlich erhöhten Ammoniakkonzentration. In der Langzeitbehandlung dieser Patienten werden die als Substrat wirkenden Aminosäuren diätetisch eingeschränkt, zudem Glycin an Phenylbutyrat oder Natriumbenzoat gebunden, um zusätzlich Stickstoff aus Aminosäuren entfernen zu können [2]. Alternativ kann die Bildung von toxischen Substraten an einer vorgelagerten Reaktion eines Stoffwechselweges blockiert werden. Dies funktioniert z. B. bei der Tyrosinämie Typ I, bei der durch Gabe des ursprünglich als Pflanzenschutzmittel entwickelten Nitisinons (NTBC) aus einer Tyrosinämie Typ I eine Tyrosinämie Typ III entsteht, die unter Einhaltung von tyrosinarmer Ernährung in den meisten Fällen einen sehr guten Verlauf nimmt [23].

#### Toxischer Metabolit bei einer Doppelfunktion von Enzymen/„metabolite repair“ (Beispiel SLC37A4-Defekt, Empagliflozin/„drug repurposing“)

**Figure 2:**
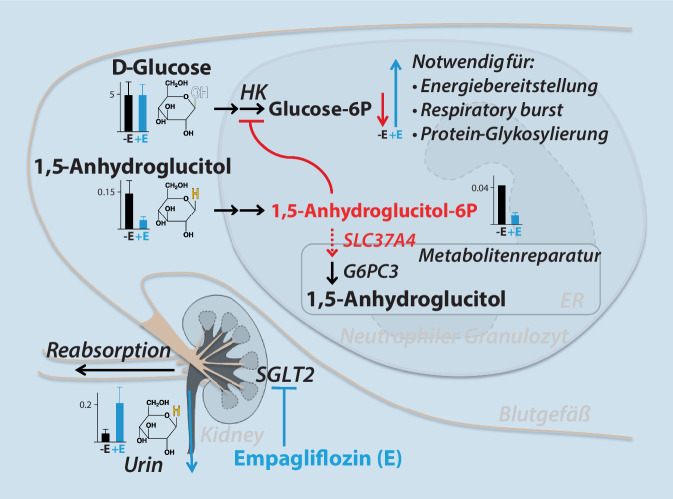

Bei der Glykogenspeicherkrankheit (GSD) Typ Ib ist nach dem Glykogenabbau die Freisetzung von Glucose aus der Leber blockiert, weil der Transporter SLC37A4 nicht funktioniert. Dieses Problem lässt sich durch häufige stärkehaltige Mahlzeiten lösen, die eine kontinuierliche Verfügbarkeit von Kohlenhydraten sichert und somit Hypoglykämien sowie dadurch verursachte neurologische Schäden abwendet. Bei einem Großteil der Patienten mit GSD Typ Ib treten aber trotz gut eingestellter Diät immunologische Probleme aufgrund einer Neutropenie mit schlecht heilenden Wunden, v. a. im Mund- und im Genitalbereich, sowie chronisch entzündlicher Darmerkrankung auf. Bis vor Kurzem war dieser Aspekt des SLC37A4-Mangels unklar, v. a., weil bei der ähnlichen GSD Typ Ia keine Neutropenie auftritt. In einer rezenten Arbeit konnte aber gezeigt werten, dass SLC37A4 eine Doppelfunktion hat und neben dem Glucose-6-phosphat-Transport auch den Transport von 1,5-Anhydroglucitol-6-phosphat (AG6P) katalysiert [27]. Das AG6P stört die Funktion von Neutrophilen und verursacht die Neutropenie bei GSD Typ Ib. Es entsteht aus 1,5-Anhydroglucitol (AG), einem metabolischen Irrläufer, der große Ähnlichkeit zu Glucose hat und mit einem geringen Anteil in Kohlenhydraten vorkommt. Das Medikament Empagliflozin, ein Inhibitor des „sodium glucose transporter 2“ (SGLT2), wird bei Diabetes mellitus eingesetzt, um eine überhöhte Glucosekonzentration durch die Ausscheidung über die Nieren in den Harn zu reduzieren. Empagliflozin kann auch zur Reduzierung der AG-Konzentration verabreicht werden, wobei (*beachte*) im Fall eines SLC37A4-Defekts aber Patienten mit dem Risiko für eine Hypoglykämie mit einer Substanz behandelt, die zur Reduktion von Glucose führen kann (Abb. 2). In individuellen Heilversuchen mit Empagliflozin bei 4 Patienten mit GSD Typ Ib konnte allerdings festgestellt werden, dass v. a. die Konzentration von AG und insbesondere AG6P reduziert wird und es zu keiner nennenswerten Hypoglykämie kommt. Durch Reduktion der toxischen Substanz AG6P wurde die Neutrophilenfunktion merklich verbessert, und die chronisch entzündlichen Prozesse konnten innerhalb weniger Wochen gestoppt werden [29].

#### Kofaktorabhängige Enzyme und Defekte im Kofaktormetabolismus Tetrahydrobiopterin – Phenylalaninhydroxylase, Riboflavin – ACAD9, Riboflavin – *FLAD1*-Defekt, Thiamin – TPK1-Mangel)

Es gibt über 30 verschiedene Kofaktoren; dies sind meist relativ kleine organische Moleküle oder Metallionen, die für die Reaktivität zahlreicher Enzyme von entscheidender Bedeutung sind. Bei Enzymdefekten, bei denen eine gewisse Restfunktion besteht, kann im Fall von kofaktorabhängigen Enzymen, eine höhere Kofaktorkonzentration zu einer höheren Restfunktion des betroffenen Enzyms führen. Ein bekanntes Beispiel ist die Erhöhung der Restfunktion der Phenylalaninhydroxylase durch hohe Konzentrationen des Kofaktors Tetrahydrobiopterin (BH_4_), v. a. bei Patienten mit einer milden Form einer PKU. Ein ähnlicher Zusammenhang konnte kürzlich für ACAD9, einen „Assembly“-Faktor des Komplex I der Atmungskette, gezeigt werden; hier erzielt die Gabe von Riboflavin ein signifikant besseres Outcome [20]. Auch bei Defekten im Metabolismus von Kofaktoren kann eine Substitution der Vorläufersubstanz die klinischen Symptome verbessern, z. B. die Thiaminsupplementierung beim TPK1-Mangel (Thiaminpyrophosphokinase, [17]) oder die Riboflavinsupplementierung beim *FLAD1*-Defekt (FAD-Synthase, [19]).

#### Enzymersatztherapie (z. B. intrazellulär für lysosomale Defekte, im Blut Phenylalanin-Ammoniak-Lyase/Pegvaliase – Phenylketonurie)

Ein sehr allgemeiner Ansatz bei einem Enzymdefekt ist die Enzymersatztherapie. Dabei wird fehlendes Protein z. B. in den Kreislauf freigesetzt, und die fehlende Funktion kann damit wiederhergestellt werden. Sehr häufige Anwendungen sind etwa Insulin bei Diabetes oder Wachstumshormon bei Minderwuchs. Bei Defekten in Zellorganellen sind v. a. lysosomale Defekte gut über eine Enzymersatztherapie ansteuerbar, da ein rekombinantes Enzym über die Endozytose in die Lysosomen transportiert wird und dort die fehlende Funktion ersetzen kann, wie z. B. einen Glukozerebrosidase-Defekt bei M. Gaucher oder einen Saure-Maltase-Mangel bei M. Pompe.

Ein Problem stellt grundsätzlich die Blut-Hirn-Schranke dar, wodurch neurologische Formen von lysosomalen Defekten weniger gut ansprechen. Auch der komplette Funktionsverlust eines betroffenen Enzyms kann problematisch sein, da ein rekombinantes Ersatzenzym häufig vom Immunsystem als Fremdstoff eingestuft wird und Antikörper gegen dieses Enzym gebildet werden, wodurch die Wirkung der Therapie weitgehend verloren geht. Einen interessanten Ansatz stellt die Verwendung einer alternativen enzymatischen Reaktion dar, in der z. B. erhöhtes Phenylalanin durch die Phenylalanin-Ammoniak-Lyase (Pegvaliase), ein Enzym aus Cyanobakterien, zersetzt wird. Für Erwachsene mit PKU wurde dieser Enzymersatz, der gespritzt werden muss, kürzlich auch in Europa zugelassen. Trotz der Polyethylenglykolmodifikation dieses Enzyms kommt es bei den meisten Patienten allerdings zu einer Immunreaktion und nur begrenzter Verwendbarkeit des Enzymersatzes.

### Stoffwechsel‑/Allgemeinerkrankungen

#### Transplantation (z. B. HSCT und Lebertransplant bei MNGIE)

Vor allem bei organspezifischen, schweren Defekten kann eine Organtransplantation eine Therapieoption darstellen. Lebertransplantationen erfolgten in etlichen Fällen bei Patienten mit Harnstoffzyklusdefekten. Neuere Beispiele für eine Lebertransplantation sind etwa Patienten mit einem Thymidinphosphorylase(TYMP)-Mangel und *m*itochondrialer *n*euro*g*astro*i*ntestinaler Enzephalopathie (MNGIE). Beim TYMP-Mangel kommt es zur toxischen Anhäufung von Nukleosiden (Thymidin and Desoxyuridin) im Plasma, zu einer Imbalance im Nukleotid-Pool und infolgedessen zur Schädigung der mitochondrialen DNA, die meist gastrointestinale und neurologische Symptomen im Kindes‑, Jugendlichen- oder jungen Erwachsenenalter auslöst und häufig sehr spät erkannt wird. Im Jahr 2005 wurden erstmals MNGIE-Patienten mithilfe der allogenen hämatopoetischen Stammzelltransplantation (HSCT) therapiert, wodurch die metabolische Auffälligkeit korrigiert und ein Fortschreiten der Krankheit verhindert werden konnte [8]. Nachteil der HSCT ist die relativ hohe Mortalität über 50 %. Als Alternative zur HSCT wurde 2014 erstmals eine Lebertransplantation beschrieben. Die Wirksamkeit der Lebertransplantation scheint auch gut zu sein, und in einer rezenten Arbeit wurde von 4 weiteren lebertransplantierten MNGIE-Patienten berichtet, von denen keiner verstorben ist [16].

### Allgemeinerkrankungen

#### Thermolabile mutierte Proteine (NBAS-Defekt und fieberinduziertes Leberversagen)

Ein Beispiel für ein relativ häufiges Auftreten von temperaturlabilen Mutationen in einem Protein stellt der NBAS-Defekt dar. NBAS ist am retrograden Transport vom Golgi-Apparat zum endoplasmatischen Retikulum beteiligt, und ein biallelischer Funktionsverlust bedingt ein wiederkehrendes akutes Leberversagen („recurrent acute liver failure“, RALF), mit häufig erstmaligem Auftreten im Säuglingsalter. Bei diesen Patienten konnte beobachtet werden, dass ein Leberversagen immer mit fieberhaften Infektionen einhergeht, und im Zellkulturexperiment kam es nach Inkubation von Patienten-Fibroblasten bei 40 °C zu einem spezifischen Verlust des NBAS-Proteins [25]. Seit der Erstbeschreibung 2015 konnten mittlerweile mehr als 100 Patienten identifiziert werden, von denen ein Großteil von einer frühzeitigen antipyretischen Therapie profitiert, unter der ein Leberversagen verhindert werden kann und eine gute Entwicklung dieser Patienten trotz ihres NBAS-Defekts möglich ist [26].

#### Somatische Gentherapie (SMA – Zolgensma, Epidermolysis bullosa – transgene Stammzellen)

Ohne eine spezielle Kenntnis des Pathomechanismus zu besitzen, könnte man sagen, dass jeder genetische Defekt durch eine Gentherapie behandelbar sein müsste. Das trifft bei einer Funktionsverminderung von Genprodukten grundsätzlich zu, allerdings gibt es – zumindest derzeit – noch erhebliche Grenzen der Machbarkeit, und erste Anwendungen beim schweren kombinierten Immundefekt (SCID) um die Jahrtausendwende brachten erste Ernüchterung, da einige behandelte Patienten entweder verstarben oder Tumoren entwickelten. Generell ist bei einer Auswahl zur Behandlung mithilfe einer Gentherapie zu sagen, dass Krankheiten, bei denen nur einzelne Organe betroffen sind, besser geeignet sind. Ende 2017 wurde als erste Gentherapie für eine erbliche Krankheit Luxturna zur Behandlung der Leberschen kongenitalen Amaurose aufgrund eines *RPE65*-Gendefekts von der Food and Drug Administration (FDA) zugelassen. Noch mehr Aufmerksamkeit hat 2019 die Zulassung einer Gentherapie der SMA mit Zolgensma hervorgerufen, nicht zuletzt auch deshalb, weil es sich mit einem Einführungspreis von über 2 Mio. USD um das bisher teuerste Medikament handelt.

Neben den bislang zugelassenen Gentherapien existieren individuelle Gentherapieansätze, wie etwa die Regeneration der gesamten Epidermis mit transgenen Stammzellen bei einem Patienten mit Epidermolysis bullosa [9]. Weitere Erfolge und Durchbrüche sind auf dem Gebiet der Gentherapie zu erwarten. Eine schnelle Lösung mithilfe der Gentherapie für alle genetischen Krankheiten ist aber unrealistisch, und sowohl die Kosten als auch die Nebenwirkungen müssen sorgfältig mit der Wirkung abgewogen werden.

#### Transkripttherapie (Spleißen, Nusinersen – *SMN1* vs. *SMN2*, Milasen – *CLN7*)

Eine interessante Alternative zur somatischen Gentherapie stellt die Transkripttherapie dar. Dabei kann auf der RNA-Ebene z. B. durch Verwendung von relativ kleiner „Anti-sense“-RNA die Expression von Genen bzw. deren Spleißen beeinflusst werden. Nusinersen (Spinraza®) ist das Beispiel eines 2016 zur Behandlung der SMA zugelassenen Medikaments, bei dem das Vorliegen des intakten *SMN2*-Gens genutzt wird, das allerdings aufgrund einer Punktmutation im Exon 7 größtenteils falsch gespleißt wird und daher nur mit sehr geringer Ausbeute in einem funktionellen SMN-Protein resultiert. Durch Einsatz des passenden Antisense-Oligonukleotids wird das Spleißen der *SMN2*-RNA verbessert und das daraus resultierende Protein kann das Fehlen von SMN1 ausgleichen [13]. Der Vorteil, dass die Therapie auf Transkriptebene nicht permanent wie bei der Gentherapie ist, ist gleichzeitig aber auch ein Nachteil, v. a. auch deshalb, weil das Antisense-Oligonukleotid im Fall der SMA intrathekal appliziert werden muss.

Vor Kurzem wurde erstmals eine maßgeschneiderte Antisense-Oligonukleotid-Therapie („Milasen“) für eine einzige Patientin mit *CLN7*-assoziierter neurodegenerativer Erkrankung („Batten disease“) erfolgreich entwickelt und innerhalb eines Jahres verabreicht. Hierunter kam es zu einer Verminderung der epileptischen Anfälle [14]. Es ist davon auszugehen, dass die derzeit milliardenfache Anwendung der RNA-Impfstoffe gegen das „severe acute respiratory syndrome coronavirus 2“ (SARS-CoV-2) der Therapie auf Transkriptebene einen Entwicklungsschub geben wird, der auch der Entwicklung von Therapien für vererbte Krankheiten helfen sollte.

## Gesundheitsökonomische Aspekte: Therapiekosten

Die Therapie von genetischen Krankheiten muss entgegen der gängigen Wahrnehmung nicht teuer sein. In einigen Fällen reicht nur eine besondere Wachsamkeit, wie im Fall eines β‑Oxidationsdefekts aufgrund der Defizienz der „medium-chain acyl-coenzyme A dehydrogenase“ (MCAD), bei dem längeres Fasten vermieden werden muss, oder beim erwähnten *PPA2*-Defekt, bei dem Betroffene keinen Alkohol konsumieren dürfen. Auch eine frühzeitige antipyretische Behandlung, z. B. beim *NBAS*-Defekt, stellt keinen nennenswerten Kostenfaktor dar. Dies gilt ebenso für die angesprochenen Therapien mit Vitaminen und anderen Nahrungsergänzungsmitteln wie z. B. Uridin oder Fucose. Auch die Behandlung der Neutropenie bei GSD Typ Ib mit einem SGLT2-Inhibitor ist um den Faktor 25 kostengünstiger als die Gabe von (hier wenig wirksamem) Granulozyten-Kolonie stimulierendem Faktor (G-CSF), und die orale Anwendung ist wesentlich patientenfreundlicher als die s.c.-Injektion. Andererseits gibt es auch die Gentherapie des SMA mit dem derzeit teuersten Medikament. Das heißt, die Kosten einer Behandlung müssen nicht hoch sein, die Kosten bei Nichtbehandlung können aber beträchtlich sein, nicht eingerechnet das Leid der Betroffenen und ihrer Familien.

## Früherkennung von behandelbaren genetischen Krankheiten

Die Erkenntnis, dass eine phenylalaninreduzierte Ernährung zu einer normalen Entwicklung bei Personen mit einem *PAH*-Defekt führen kann, war vor ca. 60 Jahren der Auslöser für das Neugeborenenscreening.

In der Zwischenzeit wurde die Liste der Krankheiten, nach denen bei Neugeborenen gesucht wird, auf ca. 10 bis 30 Krankheiten erweitert; allein in Europa bestehen bemerkenswert große Unterschiede. Ein Großteil der gesuchten Krankheiten sind Stoffwechseldefekte, nach denen über einen geeigneten Metaboliten aus Blutstropfen eines Trockenblutkärtchens gesucht wird. Über die Jahre wurde die Liste der Krankheiten angepasst, wobei v. a. die Entwicklungen im Bereich der Massenspektrometrie dem Neugeborenenscreening um die Jahrtausendwende einen Schub gegeben haben.

Die neuen Therapieoptionen bei Patienten mit SMA haben jetzt Bewegung in Richtung eines genetischen Neugeborenenscreenings gebracht. Durch ein genetisches Neugeborenenscreening könnte auch eine Vielzahl anderer genetischer Krankheiten nachgewiesen werden, bei denen eine Therapieoption besteht. Kürzlich wurde ein Update einer Liste von behandelbaren genetischen Krankheiten mit geistiger Retardierung publiziert; es sind Defekte in 139 Genen gelistet [10]. Etliche dieser Krankheiten würden die Kriterien des American College of Medical Genetics (ACMG) sowie von Wilson und Jungner für ein Neugeborenenscreening erfüllen [4]. Letztlich wird es zum Wohl der Neugeborenen mit behandelbaren genetischen Krankheiten ein Überdenken des derzeitigen Screeningansatzes brauchen. Die notwendigen Entscheidungen sind nicht einfach, da damit eine möglichst geringe Belastung für die Probanden verbunden sein soll und auch die Kosten für das Gesundheitssystem zu beachten sind. Es sollte aber das Mögliche zum Wohle der Patienten getan werden, und wir sollten auch den Mut haben, es zu tun.

## Fazit für die Praxis

- Zumindest für einen Teil der genetisch bedingten Krankheiten existieren bereits jetzt Therapieoptionen.
- Die Kenntnis der pathobiochemischen Grundlagen und Auswirkungen einer genetisch bedingten Krankheit ermöglicht die Entwicklung von gezielten therapeutischen Maßnahmen („personalisierte Präzisionsmedizin“).
- Bei Stoffwechseldefekten gibt es Therapieoptionen aufseiten des Substrats, des Produkts oder des Enzyms.
- Die wirksame Therapierbarkeit von Krankheiten muss auch zu einer Weiterentwicklung bei der Früherkennung führen, um bestenfalls einen Krankheitsausbruch gänzlich verhindern zu können.
